# Supplementary material for: Dynamic basis of lipopolysaccharide export by LptB2FGC
Source: eLife. 2024 Oct 7;13:RP99338. doi: 10.7554/eLife.99338 (PMC11458178; doi:10.7554/eLife.99338)
Supplement: Figure 2—source data 1. [file elife-99338-fig2-data1.docx]

| Variant | Protein [µM] (±10%) | Spin [µM] (±10%) | Labelling efficiency [%]  (±15%) |
| --- | --- | --- | --- |
| B_M134C | 60 | 125 | 104 |
| F_A45C – G_I335C | 37 | 72 | 113 |
| F_L325C – G_A52C | 37 | 69 | 93 |
| F_S186C – G_V209C | 31 | 70 | 113 |
| F_S156C – F_I234C | 70 | 130 | 93 |
| F_S186C – F_I234C | 31 | 61 | 98 |
| G_L152C – G_V209C | 46 | 87 | 95 |
| G_V209C – G_L234C | 34 | 64 | 94 |
| F_A45C – G_I335C + LptC | 29 | 58 | 100 |
| F_L325C – G_A52C + LptC | 31 | 62 | 100 |

**Figure 2-source data 1. Spin labelling efficiency for the investigated cysteine variants.** A 10% error is estimated for the protein and spin concentrations.
