# Supplementary figures and images for: Dynamic basis of lipopolysaccharide export by LptB2FGC
Source: eLife. 2024 Oct 7;13:RP99338. doi: 10.7554/eLife.99338 (PMC11458178; doi:10.7554/eLife.99338)

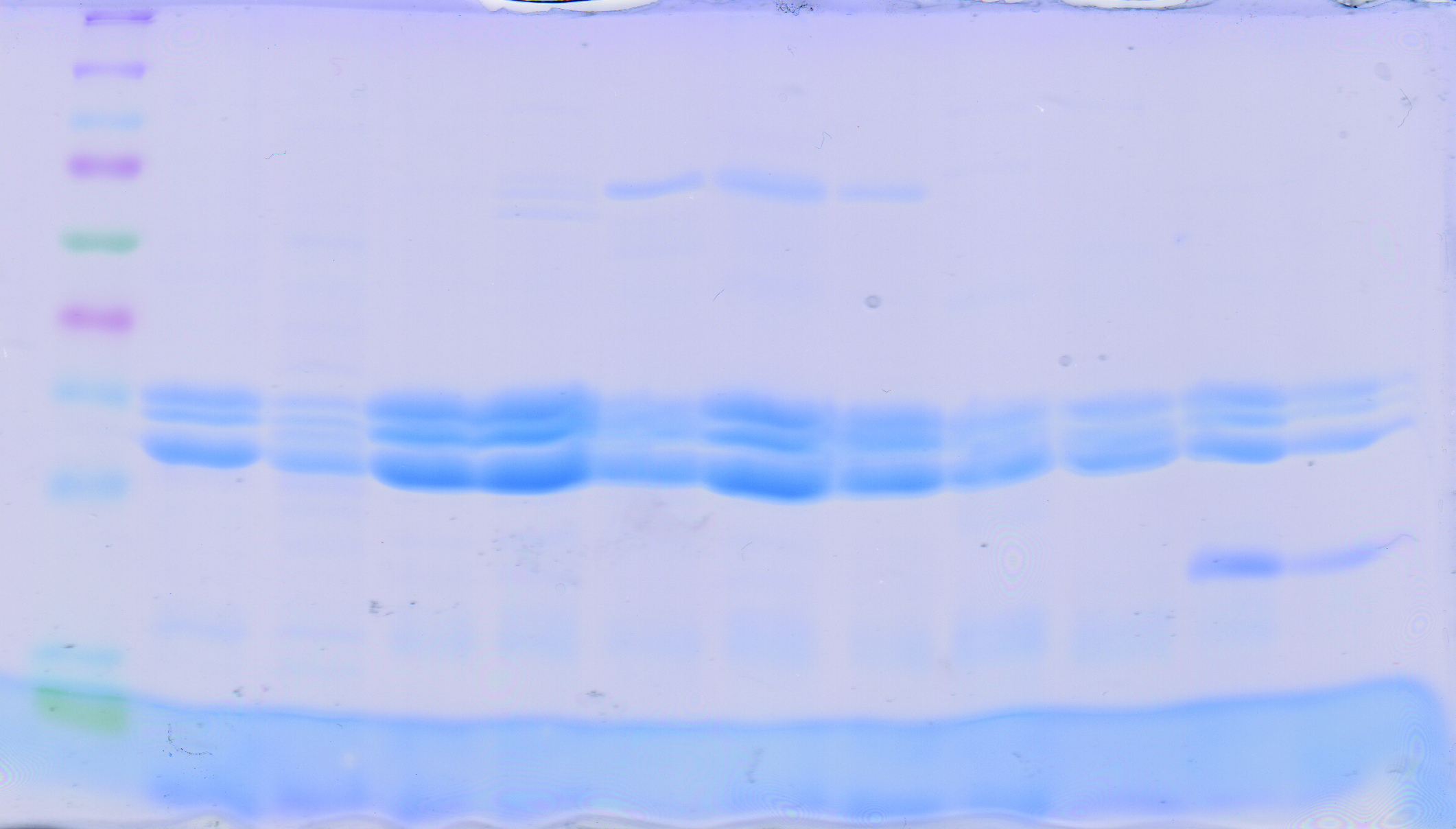

Supplement: Figure 2—figure supplement 2—source data 1. [file elife-99338-fig2-figsupp2-data1.tiff]

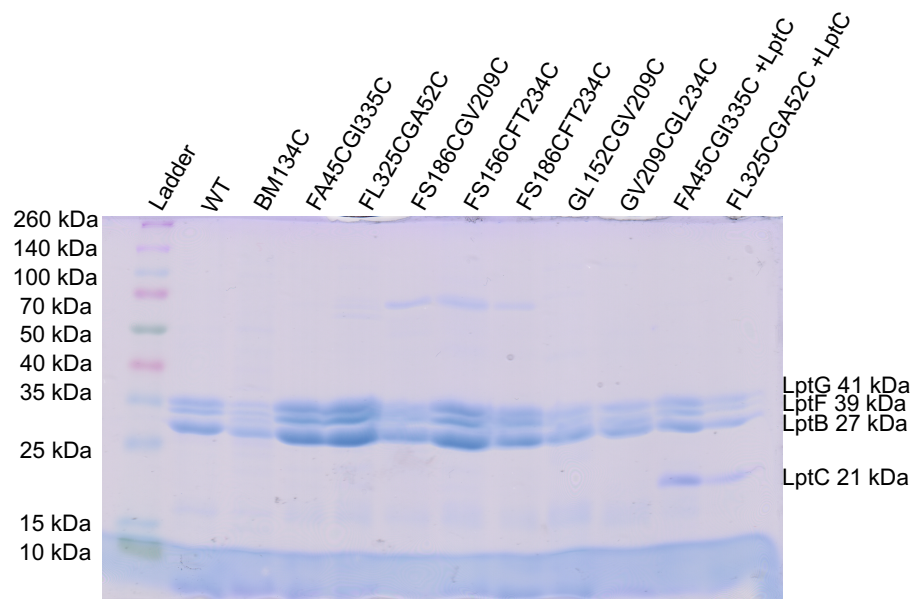

Supplement: Figure 2—figure supplement 2—source data 2. [file elife-99338-fig2-figsupp2-data2.pdf]
